# SARS-CoV-2 Infection in Kidney Transplant Recipients: A Single-Centre Study of 20 Cases from India

**DOI:** 10.1155/2021/2243095

**Published:** 2021-11-05

**Authors:** Ravi Raju Tatapudi, Venkateswara Rao Kopparti, Anusha Poosapati, Srinivas Metta, Atchyutha Rao Gongada, Balakrishna Vedulla

**Affiliations:** ^1^Apollo Hospitals, Health City, Arilova, Chinagadali, Visakhapatnam, Andhra Pradesh 530040, India; ^2^GITAM Institute of Medical Sciences and Research, Rushikonda, Visakhapatnam, Andhra Pradesh 530045, India

## Abstract

**Introduction:**

The second wave of COVID-19 has spread across India causing unprecedented misery to people since March 2021. Kidney transplant recipients (KTRs) are at an increased risk of severe infection. Their outcomes appear to be worse than those in the general population. There is no robust evidence or consensus to support any form of treatment protocol or modification of immunosuppression in KTRs with COVID-19. There is a need to develop effective and safe therapeutic protocols for this frail population. Remdesivir is the only approved antiviral drug in COVID-19 till now.

**Methods:**

We describe clinical features, role of HRCT, therapeutic protocols, and mortality rate of 20 KTRs with SARS-CoV-2 infection.

**Results:**

Complete recovery was seen in 8 (40%) patients monitored at home. 12 (60%) patients with HRCT scores more than 8/25 were hospitalized. 11 (55%) had hypoxia, of these 8 (40%) had mild hypoxia, 1 (5%) required NIV, and 2 (10%) needed mechanical ventilation. Immunosuppression was modified in all the patients. Remdesivir and dexamethasone were administered to the hospitalized patients. 1 (5%) patient had AKI requiring RRT. 1 (5%) patient expired, and 1 still hospitalized. 10 of the hospitalized patients recovered. Out of the total 20 patients, 18 (90%) recovered completely within two weeks of infection.

**Conclusion:**

Clinical presentation of COVID-19 in KTRs was similar to nontransplant patients. Early hospitalisation and assessing the severity by HRCT were important. Continuing tacrolimus and administering remdesivir and dexamethasone reduced the incidence of renal failure and improved survival rates.

## 1. Introduction 

Severe acute respiratory syndrome corona virus 2 (SARS-CoV-2) causing COVID-19 has resulted in a global pandemic since March 2020 [1]. In India, after the first case was reported in Kerala state, COVID-19 has rapidly spread across the country, and the first wave lasted till November 2020. After a lull, there was again a rapid surge in the number of cases from February 2021, reaching an unprecedented number of more than 400,000 cases and 4000 deaths per day. There was a significant increase in mortality as the second wave spiralled out of control. An avalanche of COVID-19 cases choked the health care system and crippled infrastructural facilities such as oxygen supply and availability of beds in hospitals. The second wave in India is due to the corona virus variant B-1.617 [2]. The overall fatality rate in southern states of India was 2.06% during the first wave [3]. Mortality was higher in patients with comorbidities. The most prevalent comorbid conditions among decedents were diabetes (45%) and hypertension (36.2%) [3]. In fatal cases between the ages of 18 and 29 years, renal disease was more prevalent [3].

Kidney transplant recipients (KTRs) constitute a special group and are considered to be more susceptible to SARS-CoV-2 infection and are at an increased risk of complications and mortality. This is due to chronic immunosuppressive therapy and associated comorbidities such as hypertension, diabetes, chronic kidney disease (CKD), and coronary artery disease [3, 4]. There is paucity of data regarding incidence, clinical picture, and outcome of COVID-19 among KTRs. Initial results from USA, Italy, and Spain reported a mortality of 20–28% in hospitalized KTRs [5–8]. There is no such published data in India regarding KTRs during the second wave and in those who received vaccination.

Effective treatment for COVID-19 is currently unknown [5], and there are no standardized guidelines for the treatment of infection and modification of immunosuppression regimens in these individuals. In this study, we reported the clinical features, management strategies, and outcomes of KTRs affected by COVID-19 during the ongoing pandemic. To the best of our knowledge, this is the first study from India where uniform diagnostic and treatment regimens were adopted for the management of KTRs with COVID-19 during the second wave. Primary outcomes of our study were efficacy and treatment safety, recovery, and mortality rates. Secondary outcomes of the study were necessity and mode of oxygen therapy and duration of hospital stay.

## 2. Materials and Methods

Kidney transplant recipients who had COVID-19 during the months of March, April, and May 2021 were included in this cohort study. These patients underwent transplantation at our centre within the last 22 years. Diagnosis of COVID-19 was confirmed by real-time reverse transcriptase polymerase chain reaction (RT-PCR) from nasopharyngeal swab. Detailed history and comorbidities were recorded. Clinical symptoms, temperature, and SpO_2_ were noted in all the patients. Renal function tests, complete blood counts, liver function tests, LDH, ferritin, C-reactive protein, D-dimers, procalcitonin, and IL-6 were performed once in four days. Tacrolimus trough levels were checked periodically. HRCT of the chest was done at admission and subsequently depending on the clinical need. High-resolution computed tomography (HRCT) severity score was reported by the radiologist in the hospital. We adopted COVID-19 Reporting and Data System (CO-RADS) for assessing pulmonary involvement on unenhanced chest CT images and 25-point CT severity score or total severity score (TSS) method. Basing on the severity score, we divided our patients into three groups. Mild severity was TSS less than 8/25, moderate severity was (9–18)/25, and (19–25)/25 was severe [9–11].

Patients with mild clinical symptoms of low-grade fever, cough, minor changes in haematological parameters and inflammatory markers, and normal SpO_2_ were monitored at home and were treated with paracetamol, doxycycline, and vitamins. All 20 patients were continued on their earlier immunosuppressive medication with the following modification. We continued earlier maintenance dose of prednisolone, and tacrolimus dosage was adjusted by monitoring trough levels and maintaining at 4–6 ng/ml. Antimetabolites (mycophenolate mofetil sodium or azathioprine) were reduced by half or completely withdrawn in some patients.

Patients who clinically deteriorated during home treatment experienced breathlessness and had SpO_2_ less than 92% or showed a TSS higher than 8/25 on HRCT were hospitalized and treated by the following regimen. They were closely monitored for body temperature and SpO_2_ every 6 hours. All patients received supplemental doses of vitamins. Antibiotic use was limited to the patients with secondary infection. They were given remdesivir 200 mg on the first day followed by 100 mg for the next four to seven days and dexamethasone 6 mg intravenously for ten days. No dose adjustment was made for remdesivir, as all the patients had eGFR above 30 ml/min/1·73 m^2^. We did not use methyl prednisolone, lopinavir/ritonavir, hydroxychloroquine (HCQ), or azithromycin in any of our patients.

Prophylactic anticoagulation with enoxaparin 40 mg/day was administered subcutaneously for all those with elevated D-dimer levels. Following enoxaparin for 7–10 days, they were switched to oral anticoagulants. Basing on the oxygen requirement, they were given oxygen with non-rebreather mask (NRBM), high-flow nasal catheter (HFNC), or noninvasive ventilation (NIV). Oxygen saturation was maintained above 92% in all patients.

## 3. Results

In this retrospective cohort study, we studied the clinical course and outcome of 20 kidney transplant recipients who had SARS-CoV-2 infection from March to May 2021. Infection in these patients was related to community exposure. The exact source and the time of exposure were unknown. The patients were conversant with COVID-19 appropriate behaviour. All had access to the nephrologist as they were under regular follow-up after transplantation.

All KTRs in the study underwent kidney transplantation at our centre within the last 22 years. Median time interval from transplantation to infection was 3 years. Period after transplant surgery was less than 3 months in 1, 1–10 years in 14, 10–20 years in 3, and more than 20 years in 2 patients. The time between onset of symptoms and presentation to the hospital was 2-3 days. Median age of patients in our cohort was 44 years. 10 (50%) patients were above 45 years, and 10 (50%) were below 45 years. The youngest was 9 years old, and the oldest patient was 63 years old. Male patients were 17 (85%), and females were 3 (15%). Four patients received both the doses of COVID-19 vaccination, and three received single dose (Table 1).

Comorbidities were present in all 20 (100%) patients. These included hypertension in 20 (100%), diabetes mellitus in 6 (30%), coronary artery disease in 2 (10%), CKD in 6 (30%), hepatitis C in 1 (5%), and BK virus nephropathy in 2 (10%). 8 (40%) patients had multiple comorbidities. In terms of kidney function, serum creatinine levels at the time of admission was at a median of 1·35 mg/dl.

1 patient had de novo DSA positive antibody-mediated rejection 3 months ago. She received 2 doses of intravenous immune globulin (IVIg), each 75 grams and 1 dose of rituximab. 2 patients had BK virus nephropathy. Their immunosuppression was minimized, and both had mild renal insufficiency with serum creatinine of 1.6 mg/dl.

Clinical symptoms were fever and cough in all the 20 (100%) patients, myalgias in 10 (50%), rhinorrhoea in 12 (60%), dyspnoea in 12 (60%), and diarrhoea in 6 (30%). 10 (50%) patients had loss of taste and smell (Table 2). There was lymphopenia in 11 (55%) patients with a median at 962·5 cells/cu.mm, elevated CRP in 15 (75%) with a median at 30·1 mg/ml, elevated D*-*dimer in 6 (30%) with a median at 1090 ng/ml, elevated IL-6 in 10 (50%) with a median at 16·5 ng/ml, elevated ferritin in 9 (45%) with a median at 357·8 ng/ml, and elevated LDH in 14 (70%) at 294·5 U/L. Procalcitonin greater than 0·9 ng/ml was noted in 1 (5%) patient (Table 3).

HRCT was done in all patients. 12 patients with HRCT severity scores of >8/25 were admitted in the hospital. Of these, 8 had a TSS of (8–18)/25, 4 had a TSS of more than 18/25, and all had SpO2 <90 requiring oxygen supplementation (Table 4).

All patients were continued on their regular antihypertensive and antidiabetic drugs. Tacrolimus was given in the same doses as before. 12 patients were started on dexamethasone 6 mg intravenously once daily. Remdesivir was given at a dose of 200 mg intravenously on the first day followed by 100 mg intravenously daily. 10 (84%) out of 12 patients received 5 doses of remdesivir. 2 patients received 8 doses of remdesivir. Single dose of tocilizumab 400 mg was given intravenously to 3 patients with high levels of IL-6 and hypoxia. Of the 3 patients, 1 recovered, 1 died, and 1 is on mechanical ventilation undergoing therapy in the intensive care unit. 12 patients with elevated D-dimer received enoxaparin 40 mg/day.

12 patients developed hypoxia with SpO2 <90 and were given oxygen supplementation. Out of which, 8 patients required oxygen with mask and 4 had escalated oxygen requirement who were treated with NRBM or HFNC. 2 of these patients worsened and were later intubated and mechanically ventilated.

10 patients had stable renal function throughout their hospital stay. 1 patient developed prerenal uraemia, serum creatinine increased to 3.6 mg/dl, and following correction of volume depletion, serum creatinine came down to 1·5 mg/dl. 1 patient who was treated for recent antibody-mediated rejection developed renal failure while on mechanical ventilation and required renal replacement therapy (RRT).

Mortality in the total cohort was 5% (1 out of 20). One hospitalized patient expired on the 6^th^ day while on mechanical ventilation. He had coronary artery bypass graft (CABG) 5 years ago and had severe left ventricular (LV) dysfunction. 8 patients were discharged by 8 days, 2 patients were discharged by 14 days, and 1 is currently in the hospital. All had stable serum creatinine compared to their base line (Table 2).

## 4. Discussion

We studied clinical features, management strategies, and outcomes of 20 kidney transplant recipients with SARS-CoV-2 infection, during the second wave of COVID-19 pandemic in India. Currently, there is limited knowledge regarding the treatment of COVID-19 in KTRs, and an effective treatment for this group is unknown [5, 12]. The continuation, proper dosage, and target levels of immunosuppressive drugs are still a matter of debate. Earlier analyses have shown unfavourable outcomes and high mortality in KTRs following SARS-CoV-2 infection [5, 8, 13, 14]. Caillard et al. reported that severe infection-related mortality was significantly higher in KTRs than in nontransplant patients (17·9% vs. 11·4%, *p*=0.038) [15].

We describe the clinical course of 20 KTRs with COVID-19. All of them underwent transplantation at our centre and were on regular follow-up. There was male preponderance (85%). The overall patient profile with regard to age and comorbidities appears to be similar to other studies [13, 16]. Living-related donor recipients were 17, and deceased donor recipients were 3. A majority of the kidney transplantations in our centre are live-related donor transplants.

All patients in the cohort presented with fever and cough, and COVID-19 symptoms were similar to those in the general population where 58–100% had fever, 42–100% had cough, 5–90% had dyspnea, and 5–90% had fatigue and myalgia [13, 14, 17]. Similar presentation was described in reports of Columbia University Transplant Program in New York [6] and single-centre Italian study of COVID-19 in KTRs [18]. Though the initial presentation was benign, 3 out of 11 patients in our study deteriorated fast, requiring hospitalisation and oxygen therapy. Other studies also reported a more rapid clinical progression than in general population [4].

Lymphopenia with reduction in absolute lymphocyte count was noticed in 11 (55%) patients. Lymphopenia was reported to be 83.2% in the infected patients by Guan et al. [19]. In general population with COVID-19, lymphopenia was observed in 63% of the patients. Other studies reported an association between lymphopenia, severity of the disease, acute respiratory distress syndrome (ARDS), and fatal outcome [20]. The cause of lymphopenia is not clear although lymphocytes have been shown to be the primary targets of SARS-CoV-2. In our study, 15 patients had elevated CRP levels. Wu et al. and Deng et al., in their studies on risk factors, have shown a strong association between elevated CRP and unfavourable outcomes including ARDS and death in COVID-19 patients [21, 22]. Though the relation between high levels of ferritin and severity of illness and death is not well established, some studies have shown that higher ferritin levels are associated with ARDS. In our cohort, serum ferritin levels were elevated in five patients. And in one, it was very high, and she developed respiratory distress, thus requiring mechanical ventilation. IL-6 was found to be consistently elevated in COVID-19 patients with respiratory failure [23]. Tocilizumab was shown to be associated with significantly improved survival of COVID-19 patients and rescued them from rapid death [23]. In our cohort, three patients with hypoxia and elevated IL-6 were given tocilizumab. Of these patients, one expired, and one is on mechanical ventilation. D*-*dimer was elevated in 6 (30%) of our patients, and all of them were given anticoagulant prophylaxis with enoxaparin. None of them had thrombotic complications during or after recovery. Yang et al. showed that D*-*dimer was elevated in those with severe symptoms and possible disseminated intravascular coagulation (DIC) [18, 19, 24].

All admitted patients had CT severity scores above 8/25. Some patients in the cohort had worsening of severity score on HRCT, and their oxygen requirement increased. Earlier reports of radiological data of COVID-19 focussed on CT findings of the chest [25–27]. Chest X-ray is employed as a first-line triage tool by the British and Italian hospitals [28, 29]. British Society of Thoracic Imaging has not recommended use of CT except in seriously ill patients [30]. We found that HRCT was useful for assessing the severity of lung involvement initially and for monitoring the progress of pneumonia and its response to treatment, similar to reported radiological data of COVID-19 [25–27]. Low-dose HRCT done after treatment showed significant reduction in severity score. 12 patients in our cohort were hypoxic and had moderate to severe pneumonia as per the CT severity score at a median of 6.5 days after the onset of initial symptoms.

Azithromycin, antiretroviral drugs (oseltamivir, ritonavir, darunavir, and lopinavir), and hydroxychloroquine (HCQ) were used extensively in KTRs with COVID-19 during the first wave of pandemic [8, 31]. In fact, lopinavir/ritonavir was used as the first-line drug in several countries [32]. In contrast, LPV/r monotherapy was reported to be ineffective in hospitalized COVID-19 patients by the ‘recovery trial' [33]. Protease inhibitors will enhance serum tacrolimus levels which in turn may prolong QT interval in a dose-dependent manner leading to fatal arrhythmias. Combination of azithromycin and hydroxychloroquine may also prolong corrected QT-interval [8, 34]. Hence, the drug combinations must be handled carefully to prevent drug interactions. Several centres stopped using these drugs in the treatment against COVID-19. Acute kidney injury (AKI) has been reported in COVID-19 patients due to virus-induced direct toxicity on proximal tubule epithelial cells, dehydration, volume depletion, cytokine release syndrome, and hemodynamic changes [34]. AKI has been reported in up to 15% of COVID-19 patients in general population [35]. In KTRs with COVID-19, 21–60% of the patients were noticed to have AKI, while 10% of them required RRT [36]. Kute et al. also reported high rates of AKI (48·4%) in their patients [13]. It is interesting to note that AKI was common in those patients who received lopinavir/ritonavir (25%). In their study, Alberici et al. reported that 6 out of 20 patients developed AKI and 1 required hemodialysis [8]. We believe that the incidence of AKI in our cohort was low (5%) as we have not used antiretroviral drugs, azithromycin, or HCQ in our patients.

Immunosuppression management in KTRs with COVID-19 is found to be challenging. All our KTRs were on triple immunosuppression with prednisolone, tacrolimus, and mycophenolate mofetil or azathioprine before the onset of infection. We stopped prednisolone and mycophenolate/azathioprine in all the hospitalized patients. In our cohort, one patient developed prerenal uraemia due to severe vomiting. His renal function improved following correction of volume depletion. The patient who was treated for ABMR (antibody-mediated rejection) during the past three months developed worsening of renal function after mechanical ventilation and required RRT. Rest of the patients had stable kidney function. We feel that higher incidence of AKI in the KTR group than in the nontransplant group might be due to the toxicity of lopinavir/ritonavir, supratherapeutic levels of tacrolimus caused by drug interaction, calcineurin inhibitor (CNI) toxicity, or acute rejection due to withdrawal of immunosuppression. In their short series, Elhadedy et al. showed that hospitalized KTRs with COVID-19 can be managed conservatively with oxygen supplementation and adjusting immunosuppression by withholding mycophenolate. However, they recommended the use of remdesivir in this group of patients as we have followed in our treatment regimen [20].

Remdesivir is a nucleoside analogue that inhibits viral RNA-dependant RNA polymerase, thus interfering with RNA replication. The Adaptive COVID-19 Treatment Trial (NCT04280705) demonstrated that 31% faster recovery time can be attained in remdesivir-treated patients when compared to controls [37]. US FDA has authorized the use of remdesivir as a treatment option for hospitalized COVID-19 patients. 12 patients in our cohort received 5–8 doses of remdesivir without any untoward effects. Their hepatic and kidney function remained stable. Tacrolimus trough levels were maintained at 4–6 ng/ml, and there was no nephrotoxicity due to supratherapeutic levels. Pettit et al. described that remdesivir-associated adverse effects occurred infrequently and with low severity and were not different in those with and without severe renal insufficiency [38]. Remdesivir has not been reported to cause any liver function abnormalities or any other major adverse events [39].

The Recovery Collaborative Group has shown that dexamethasone benefits hospitalized patients receiving oxygen therapy. As all our admitted patients were high-risk group and had moderate to severe pneumonia with or without oxygen supplementation, we gave them dexamethasone as per the protocol of Recovery Trial [33].

In three previous case series and one multicentre study, [4, 5, 8, 13] where KTRs with COVID-19 were treated with antiretroviral drugs and/or HCQ and azithromycin, the mortality ranged from 14.8% to 28%. In our study, 8 patients who were telemonitored at home had uneventful course and recovered in 8–10 days. Out of 12 hospitalized patients in our cohort, 10 (91·7%) patients recovered completely, 5 patients had a hospital stay of 8 days, and 5 were discharged on the 14^th^ day. 2 patients were treated in ICU, 1 required noninvasive ventilation, and 1 required mechanical ventilation. 1 patient on NIV rapidly deteriorated in 6 hours required mechanical ventilation and expired. He had CABG 4 years prior to his kidney transplantation. Hence, we had a mortality rate of 5% in our cohort of 20 KTRs with COVID-19. The multicentre study from India reported an overall mortality of 14.8% in hospitalized patients [13]. 47% of their ICU patients and 97% of their mechanically ventilated patients expired [13]. The Columbia University Group Study reported a mortality of 25% in their cohort of KTRs with COVID-19 [5]. It will be meaningful if we compare them with mortality rates in general population with COVID-19. Mortality in our cohort of transplant recipients was comparable to nontransplant hospitalized patients.

It is very tempting to speculate the efficacy of our regimen in improving the outcomes of kidney transplant recipients with COVID-19 infection having moderate to severe pneumonia. As the immunosuppression is modulated and not completely withdrawn, there were no episodes of renal failure due to allograft rejection. All the patients had an eGFR >30 ml/min/1·73 m^2^, and remdesivir was given in normal doses.

Small sample size, short duration of the study, and limited period of follow up are limitations in the current study. The confounding factor in our study is dexamethasone, and one may attribute the benefit to dexamethasone alone.

## 5. Conclusion

We presented 20 kidney transplant recipients with SARS-CoV-2 infection. 12 of them were admitted to hospital. Their presenting symptoms were similar to COVID-19 in general population. There was rapid deterioration of some of these patients compared with COVID-19 patients in general population. HRCT was important in assessing the severity of pneumonia and decision regarding hospitalisation of patients. We believe home treatment is not safe as there are no definitive parameters to decide which of these patients are likely to develop complications requiring urgent hospitalisation. Early hospitalisation and aggressive management helped in reducing the mortality of KTRs. Uniform diagnostic approach and treatment plan has contributed to favourable outcome and recovery. Appropriate modification of immunosuppression and continuation of tacrolimus with monitoring of trough levels of the drug aided in the prevention of allograft rejection as well as CNI toxicity. Combination of remdesivir and dexamethasone therapy along with standard of care treatment appears to have significantly contributed to the reduced mortality. Larger clinical registries and randomized control studies are required to establish the efficacy of this treatment strategy in transplant recipients.

## Figures and Tables

**Table 1:** Basic clinical profile of kidney transplant recipients with SARS-CoV-2 infection.

Baseline profile	*n* (%)
Age (years)	
<18	1 (5%)
18–45	10 (50%)
45–65	9 (45%)
Total	20

Gender	
Male	17 (85%)
Female	3 (15%)

Interval after transplantation	
<10	15 (75%)
10–20	3 (15%)
>20	2 (10%)

Donor	
Live-related	17 (85%)
Deceased	3 (15%)

Comorbidities	
Diabetes	6 (30%)
HTN	20 (100%)
CKD	6 (30%)
CAD	2 (10%)
BKV	2 (10%)
HCV	1 (5%)
Multiple comorbidities	8 (40%)

Vaccination	
Two doses	4 (20%)
First dose	3 (15%)

HTN = hypertension; CKD = chronic kidney disease; CAD = coronary artery disease; BKV = BK virus; HCV = hepatitis C virus. This table depicts the basic demographics and clinical profile of KTRs included in the study. Transplantation and vaccination particulars are also included in the table. Data are represented as numbers (*n*) and percentages for each parameter.

**Table 2:** Clinical symptoms, treatment modalities, and outcomes of kidney transplant recipients with COVID-19.

Clinical symptoms	*n* (%)
Fever	20 (100%)
Cough	20 (100%)
Dyspnoea	11 (55%)
RT-PCR test COVID-19 positive	20 (100%)
Inpatient	12 (60%)
Telemedicine	8 (40%)

Immunosuppression	
Tacrolimus	20 (100%)
Mycophenolate mofetil 50% dose reduction	7/18 (39%)
Mycophenolate mofetil withheld	11/18 (61%)
Azathioprine 50% dose reduction	1/2 (50%)
Azathioprine withheld	1/2 (50%)
Remdesivir	12 (60%)
Dexamethasone	12 (60%)

Oxygen supplementation	
Mask	8 (40%)
Noninvasive ventilation (NIV)	1 (5%)
Mechanical	2 (10%)
Tocilizumab	3 (15%)

Outcome	
Recovered	18 (90%)
Died	1 (5%)
In hospital	1 (5%)

General clinical symptoms observed in KTRs with COVID-19 infection, their treatment modalities, and the consequent treatment outcomes obtained are presented. *n* values and percentages of KTRs exhibiting each parameter are shown.

**Table 3:** Laboratory findings of kidney transplant recipients with SARS-CoV-2 infection.

Laboratory tests	Entire group (*n* = 20)median (range)	Normal values median (*n*; range)	Abnormal values median (*n*; range)
Hb (g/dL)	13 (range, 8.6–18)	14.25 (*n* = 6; range, 14–14.8)	13 (*n* = 14; range, 8.6–18)
Lymphocytes (*µ*/L)	962.5 (range, 720–4200)	3800 (*n* = 9; range, 2800–4200)	850 (*n* = 11; range, 720–975)
C-reactive protein (mg/ml)	20.1 (range, 0.45–150.6)	1 (*n* = 5; range, 0.455–4)	30.1 (*n* = 15; range, 9.3–150.6)
D dimer (ng/ml)	375.5 (range, 110–>8000)	280.5 (*n* = 14; range, 110–480)	1090 (*n* = 6; range, 594–>8000)
IL-6 (pg/ml)	8 (range, 1–47.8)	2.15 (*n* = 10; range, 1–4)	16.5 (*n* = 10; range, 12–47.8)
Ferritin (ng/ml)	357.8 (range, 21–1980)	193 (*n* = 11; range, 39–384)	357.8 (*n* = 9; range, 21–1980)
Lactate dehydrogenase (U/L)	268 (range, 140–525)	183.5 (*n* = 6; range, 140–211)	294.5 (*n* = 14; range, 230–525)
SGPT (U/L)	28 (range, 16–87)	28 (*n* = 19; range, 16–47)	87 (*n* = 1)
Serum creatinine (mg/dL)	1.35 (range, 0.5–3.0)	1 (*n* = 7; range, 0.8–1.2)	1.7 (*n* = 13; range, 0.5–3.0)
Procalcitonin (ng/ml)	0.05 (range, 0.01–1.2)	0.05 (*n* = 19; range, 0.01–0.12)	1.2 (*n* = 1)

*Note.* Conversion factors for units: serum creatinine in mg/dL to *μ*mol/L × 88.4. Laboratory findings of KTRs with SARS-CoV-2 infection are detailed in this table. *n* values along with median, and ranges of each parameter are tabulated for the entire group. Median and ranges are calculated for the subgroups of KTRs exhibiting either normal or abnormal values for each of the parameter as shown in the table.

**Table 4:** HRCT scores of kidney transplant recipients with COVID-19 infection.

HRCT TSS	Median (*n*; range)
Normal	*n* = 8
Moderate	18 (*n* = 8; range, 12–18)
Severe	22.5 (*n* = 4; range, 20–23)

The total severity scores (TSS) of kidney transplant recipients with SARS-CoV-2 infection, obtained from the high-resolution chest CT scan, are presented. Data are presented as the number (*n*) of KTRs with normal, moderate, and severe TSS. Median and ranges were calculated wherever available as depicted in the table.

## Data Availability

The entire data used for analysis in this study are included in this article in the text and tables. Original patient reports cannot be made publicly available due to legal reasons. Further inquiries can be directed to the corresponding author.
